# Supplementary figures and images for: Coopted temporal patterning governs cellular hierarchy, heterogeneity and metabolism in Drosophila neuroblast tumors
Source: eLife. 2019 Sep 30;8:e50375. doi: 10.7554/eLife.50375 (PMC6791719; doi:10.7554/eLife.50375)

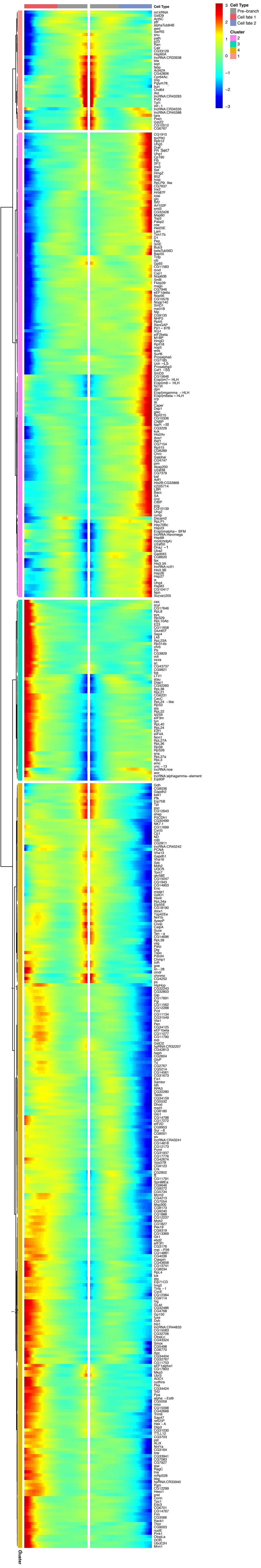

Supplement: Supplementary file 3. — Prebranch starts at the root of the trajectory. Cell fate one corresponds to the E93+stg+ branch. Cell fate two corresponds to the E93+E(spl)+ branch as labeled in Figure 7A. [file elife-50375-supp3.pdf]
